# Effectiveness of long-term treatment with SGLT2 inhibitors: real-world evidence from a specialized diabetes center

**DOI:** 10.1186/s13098-017-0297-y

**Published:** 2017-12-01

**Authors:** Yotsapon Thewjitcharoen, Nalin Yenseung, Areeya Malidaeng, Soontaree Nakasatien, Phawinpon Chotwanvirat, Sirinate Krittiyawong, Ekgaluck Wanothayaroj, Thep Himathongkam

**Affiliations:** Diabetes and Thyroid Center, Theptarin Hospital, Bangkok, Thailand

**Keywords:** Sodium-glucose co-transporter 2 inhibitors (SGLT2i), Long-term treatment, Real-world evidence

## Abstract

**Background:**

Diabetes is a progressive disease needing multiple drugs for achieving and maintaining good glycemic control. Sodium-glucose co-transporter 2 inhibitors (SGLT2i) is a novel class of anti-diabetic agent which offers several beneficial effects. However, the long-term effectiveness in clinical practice and safety data of SGLT2 inhibitors is limited, especially in Asian patients. To better understand the effectiveness of SGLT2i in clinical practice, we conducted a retrospective evaluation of patients with diabetes on SGLT2i.

**Methods:**

This retrospective observational study uses data of patients with diabetes who had been prescribed SGLT2i and continued to use at least 6 months at Theptarin Hospital, Bangkok. The characteristics of patients, changes in glycemic control and body weight at 3, 6, 12, 18, 24 months and the last follow-up were evaluated.

**Results:**

A total of 189 patients with diabetes (females 50.3%, mean age 59.9 ± 12.3 years, T2DM 97.3%, duration of diabetes 16.3 ± 9.2 years, baseline BMI 29.9 ± 6.1 kg/m^2^, baseline HbA_1c_ 8.8 ± 1.6%) were prescribed SGLT2i during the study period. At the time of first SGLT2i prescription, 80.4% used three or more other anti-diabetic agents concomitantly and 34.6% used insulin concomitantly. 151 patients who continue to use at least 6 months were included in analysis. At the last follow-up (median time 16 months), overall median HbA_1c_ reduction and weight reduction were 1.0% and 1.5 kg, respectively. While glycemic control could maintain up to 18 months, weight loss gradually rebounded after the first 6 months and then backed to baseline body weight at 18 months (78.2 ± 18.0 kg vs. 78.0 ± 17.8, p value = 0.324). The incidence of adverse drug reactions of special interest (polyuria, volume depletion-related events, urinary tract infection, genital infection, and hypoglycemia) was 2.1, 1.6, 2.1, 2.6, and 7.9%, respectively.

**Discussion:**

This real-world study confirmed long-term durability of glycemic control with SGLT2i in not only monotherapy, but also add-on studies with other oral anti-diabetic drugs and/or insulin treatment. However, weight loss became evident early after 6 weeks then reached slightly rebounds after 24 weeks until the end of follow-up. Further studies should be done towards a better understanding of treatment with SGLT2i in routine clinical practice.

## Background

The prevalence of diabetes mellitus is growing globally, reaching epidemic proportions along with the worsening of obesity worldwide [1]. Unfortunately, more than 50–70% of patients with type 2 diabetes mellitus (T2DM) failed to achieve optimal glycemic control [2, 3]. While there is ongoing debate whether strict glycemic control could benefit in prevention of macrovascular complications in diabetic patients, the established evidences strongly confirmed suboptimal glycemic control has major consequences on long-term microvascular complications [4]. The durability of oral anti-diabetic agents is typically limited by the natural history of progressive declining in β-cell function, therefore, multiple drugs are needed for achieving and maintaining good glycemic control.

Sodium-glucose co-transporter 2 inhibitors (SGLT2i) which could be used in any stage of the disease independently of existing co-medications are a promising new class of oral anti-diabetic agents [5, 6]. Since the results of the EMPA-REG OUTCOME and CANVAS trials showed that this novel anti-diabetic agent reduced the incidence of cardiovascular events in patients with T2DM and established cardiovascular disease [7, 8], there has been great interest in expanding use of SGLT2i in treating heart diseases especially heart failure [9]. Interestingly, these benefits were observed unprecedentedly with less than 1 year of treatment. Beyond glycemic control, SGLT2i offer the potential benefits of weight loss resulting from calorie loss through glycosuria and arterial blood pressure reduction associated with its diuretic effect [10, 11]. Nevertheless, some safety concerns remain, such as genital mycotic infections, urinary tract infections, risks of dehydration, risks of diabetic ketoacidosis in vulnerable patients, which repeatedly reported in studies worldwide [12–14]. Therefore, risks and benefits should be assessed on a case-by-case basis because of its specific mechanism of action.

Currently, there are at least seven types of oral SGLT2i such as canagliflozin, dapagliflozin, empagliflozin, ertugliflozin, ipragliflozin, luseogliflozin, and tofogliflozin. Moreover, several other drugs are also in different stages of development [6]. Since 2014, dapagliflozin had been approved for use in Thailand and then empagliflozin and canagliflozin were available later. According to the latest updated DM clinical practice guideline from Diabetes Association of Thailand [15], SGLT2i were positioned as part of type 2 diabetes treatment armamentarium in patients who have inadequate glycemic control by conventional drugs. It is uncertain that data from real-world studies of SGLT2i will obtain those outcomes as seen in clinical trials. To further understand SGLT2i use in clinical practice, we conducted a retrospective long-term evaluation of the metabolic parameters and adverse events in patients with diabetes on SGLT2i for at least 6 months.

## Methods

This retrospective observational study used data obtained from database of patients with diabetes who had been prescribed SGLT2i and continue to use it for at least 6 months between November 1, 2014 and June 30, 2016 in Theptarin hospital, one of the largest and most comprehensive diabetes centers in Bangkok, Thailand. The characteristics of patients, changes in glycemic control and body weight at baseline, 3, 6, 12, 18, 24 months and at the last follow-up were evaluated. The index date for each patient was the date of the first prescription fill for a SGLT2i. The existence of cardiovascular risk factors and events was determined by their presence in medical records. The effectiveness of SGLT2i in dipeptidyl peptidase-4 inhibitors (DPP4i)-treated patients compared with non DPP4i-treated patients was analyzed. The effectiveness of elderly diabetic patients who aged ≥ 65 years old were also evaluated and compared with younger patients. This retrospective study is approved by the ethics board committee of Theptarin hospital (No. 04/2016).

## Statistical analysis

Continuous variables were presented as mean (± standard deviation) and categorical variables were presented as proportions. Comparisons between DPP4i-treated patients and non DPP4i-treated patients were done using an unpaired Student’s t test in continuous data and using a Chi square test in categorical data. Comparisons between elderly patients who aged ≥ 65 years old and younger patients were also done. p value ≤ 0.05 was considered statistically significant. At the last visit, analyses of change from baseline in HbA_1c_ and body weight were performed using longitudinal repeated-measures analysis over time. All statistical analyses were conducted using the Statistical Package for the Social Sciences (version 18.0; SPSS, Chicago, IL, USA).

## Results

### Patients

**Fig. 1:**
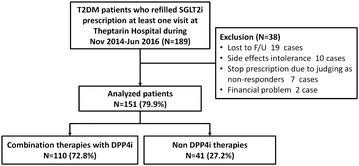
Flow diagram of analyzed patients who were treated with SGLT2i

A total of 189 patients with diabetes (females 50.3%, mean age 59.9 ± 12.3 years, T2DM 97.3%, duration of diabetes 16.3 ± 9.2 years, baseline BMI 29.9 ± 6.1 kg/m^2^, baseline HbA_1c_ 8.8 ± 1.6%) were prescribed SGLT2i during the study period. At the time of first SGLT2i prescription, 80.4% used three or more other anti-diabetic agents concomitantly and 34.6% used insulin concomitantly. Thirty-eight patients (20.1%) discontinued SGLT2i within the first 24 weeks of treatment. Failure to attend scheduled visits (19 patients, 10.1%) was the most common reason for treatment discontinuation. Other reasons included adverse events (10 patients, 5.3%), limited or no response (7 patients, 3.6%), and patient request (2 patients, 1.1%). 151 patients who continue to use at least 6 months (duration of treatment 16.9 ± 6.8 months) were included in analysis. Flow diagram of analyzed patients is shown in Fig. 1. Type of SGLT2i in our study only composed of dapaglifozin (86.1%) and empaglifozin (13.9%) because canagliflozin did not available yet during study period. Only 7.9% of prescribed patients had a history of ischemic heart disease and only 2.0% had a history of congestive heart failure at baseline. Use of SGLT2i had the neutral effects in major adverse cardiovascular events at the median time of follow-up 16 months. The details characteristics of analyzed patients are shown in Table 1.

**Table 1:** Baseline characteristics of T2DM patients who were treated with SGLT2i (N = 151)

	Total patients (N = 151)	DPP4i-treated patients (N = 108)	Non DPP4i-treated patients (N = 43)	p value
Age (years)	61.1 ± 12.2	60.7 ± 11.5	62.3 ± 14.1	0.473
Gender (% female)	79 (52.32%)	53 (48.18%)	26 (63.41%)	0.096
Duration of DM (years)	16.7 ± 9	16.3 ± 9	18 ± 9.1	0.297
Pre-treatment HbA_1c_ (%NGSP)	8.8 ± 1.5	8.9 ± 1.4	8.4 ± 1.6	0.093
Baseline BW (kg)	78.2 ± 17.8	77.6 ± 18.7	79.8 ± 15.3	0.504
Baseline BMI (kg/m^2^)	29.8 ± 6	29.4 ± 6.1	31 ± 5.5	0.142
BMI categories (%)				
< 23.0 kg/m^2^	12 (7.95%)	10 (9.09%)	2 (4.88%)	0.395
≥ 23.0– < 25.0 kg/m^2^	17 (11.26%)	15 (13.64%)	2 (4.88%)	0.130
≥ 25.0– < 30.0 kg/m^2^	56 (37.09%)	42 (38.18%)	14 (34.15%)	0.648
≥ 30.0 kg/m^2^	66 (43.71%)	43 (39.09%)	23 (56.1%)	0.061
Diabetes treatment (%)				
No oral therapy	3 (1.99%)	(0%)	3 (7.32%)	0.004
1 oral therapy	10 (6.62%)	3 (2.73%)	7 (17.07%)	0.002
2 oral therapy	30 (19.87%)	14 (12.73%)	16 (39.02%)	< 0.001
≥ 3 oral therapy	108 (71.52%)	93 (84.55%)	15 (36.59%)	< 0.001
Concomitant SU (%)	84 (55.63%)	64 (58.18%)	20 (48.78%)	0.301
Concomitant TZD (%)	74 (49.01%)	53 (48.18%)	21 (51.22%)	0.740
Insulin treatment	50 (33.11%)	33 (30%)	17 (41.46%)	0.183
Comorbid conditions (%)				
Ischemic heart disease	11 (7.28%)	8 (7.27%)	3 (7.32%)	0.765
Congestive heart failure	3 (1.99%)	1 (0.91%)	2 (4.88%)	0.026
Cerebrovascular disease	2 (1.32%)	2 (1.82%)	0 (0%)	0.565
Duration of SGLT2i (months)	17.8 ± 6.9	18.2 ± 6.7	16.9 ± 7.3	0.543

### Effectiveness

**Fig. 2:**
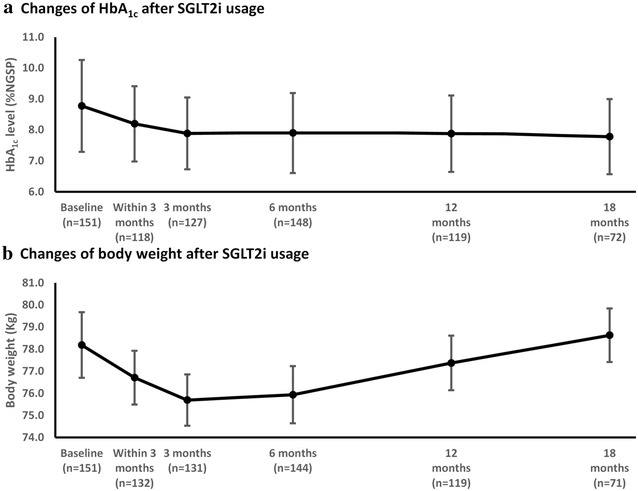
Changes in glycemic control (**a**) and body weight (**b**) over time in analyzed T2DM patients from baseline up to 18 months of follow-up period

**Fig. 3:**
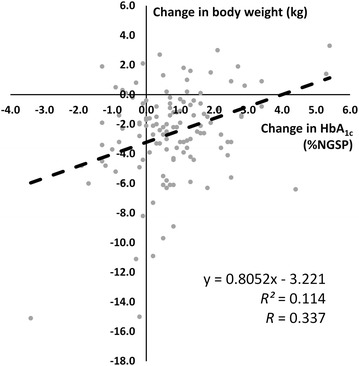
Scatter plots for the relationship between the change in HbA_1c_ and the change in body weight from baseline to the last follow-up (median time 16 months)

At 6 months, mean HbA_1c_ reduced from 8.8 ± 1.5 to 7.9 ± 1.3% and weight reduced from 78.2 ± 18.0 to 75.9 ± 17.5 kg. While glycemic control could maintain up to 18 months, weight loss gradually rebounded after the first 6 months and then backed to baseline body weight at 18 months (78.2 ± 18.0 kg vs. 78.0 ± 17.8, p value = 0.324). At the last follow-up (median time 16 months), overall median HbA_1c_ reduction and weight reduction when compared with at baseline were 1.0% and 1.5 kg, respectively. The proportion of all patients attaining HbA_1c_ target (HbA_1c_ < 7%) increased from 9.3 to 26.5% at the last follow-up. The changes of HbA_1c_ and body weight from baseline up to 18 months are shown in Fig. 2. Overall, the change in bodyweight was only weakly correlated with the change in HbA_1c_ as shown in Fig. 3.

**Fig. 4:**
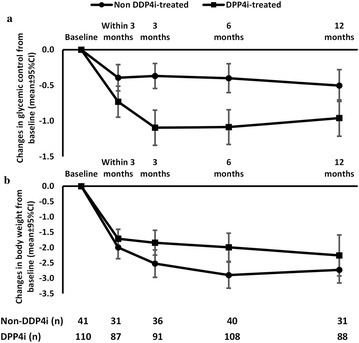
Comparison of changes in glycemic control (**a**) and body weight (**b**) over time between DPP4i-treated patients (N = 110) and non DPP4i-treated patients (N = 41)

Among DPP4i-treated patients (N = 110, baseline HbA_1c_ 8.9 ± 1.4%, median duration of DPP4i 51 months), overall median HbA_1c_ reduction and weight reduction at the last follow-up (median time 16 months) when compared with at baseline were 0.9% and 2.0 kg, respectively. While non DPP4i-treated patients (N = 41, baseline HbA_1c_ 8.4 ± 1.6%, overall median HbA_1c_ reduction and weight reduction at the last follow-up (median time 16 months) when compared with at baseline were 0.2% and 2.7 kg, respectively. Comparison of HbA_1c_ reduction between both groups from baseline up to 12 months is shown in Fig. 4.

Elderly patients (aged ≥ 65 years) composed of 53 patients (35.1%) and younger patients (aged < 65 years) composed of 98 patients (64.9%) in this analysis. At the last follow-up, median HbA_1c_ reduction (0.6 vs. 0.8%, p value = 0.608) and weight reduction (2.0 vs. 3.0 kg, p value = 0.154) were not different in elderly patients when compared with younger patients. Increased hematocrit at last follow-up when compared with baseline (2.9% vs. 3.0%, p value = 0.268) was observed frequently in both groups of patients.

### Blood pressure and other laboratory variables

The reductions in systolic blood pressure (SBP) and diastolic blood pressure (DBP) were observed from baseline to the last visit with mean SBP reduction 8.2 mmHg and DBP reduction 3.6 mmHg (133/74 vs. 125/70, p value = 0.014). The reduction in median fasting triglycerides was slightly better at the last visit (141 mg/dL vs. 137 mg/dL, p value = 0.041). There was no change in mean low-density lipoprotein cholesterol (LDL) before and after treatment (93 mg/dL vs. 92 mg/dL, p value = 0.939). Median liver enzyme (alanine aminotransferase, ALT) decreased from baseline at 27 to 20 U/L (p value = 0.026). There was no change in estimated glomerular filtration rate before and after treatment (83 mL/min/1.73 m^2^ vs. 82 mL/min/1.73 m^2^, p value = 0.572). Mean hematocrit increased from baseline at 38.9 to 41.8% at last visit (p value = 0.039).

### Adverse events

The incidence of adverse drug reactions of special interest (polyuria, volume depletion-related events, urinary tract infection (UTI), genital infection, and hypoglycemia) was 2.1, 1.6, 2.1, 2.6, and 7.9%, respectively. These events were mild or moderate in intensity, with more than 90% occurring in the first 24 weeks. UTI or genital infection occurred more frequently in women than in men. Signs or symptoms suggestive of UTI or genital infection responded to standard treatment typically without stopping of SGLT2i therapy. No event of pyelonephritis was found. No diabetic ketoacidosis (DKA) occurred during study period but one major episode of hypoglycemia after SGLT2i treatment which judged to be non-medicated related. Accumulative rate of non-severe hypoglycemia increased from 11.9% at baseline to 19.8% at the last follow-up (median time 16 months). No discontinuations were due to hypoglycemia. A comparison of total adverse events in the elderly and younger patients showed no difference between two groups of patients (9.2% vs. 10.6%, p value = 0.596).

## Discussion

Despite advancement in the treatment of T2DM, optimal glycemic control has not been achieved in almost half of patients in the US [2] or almost 70% of patients in Thailand [3]. Sodium-glucose co-transporter 2 inhibitors (SGLT2i) are the newest therapeutic option available for the treatment of T2DM which arrived into Thailand market at the end of 2014. The insulin-independent effect of this novel anti-diabetic agent is associated with a low risk of hypoglycemia, which makes it attractive for the management of patients with T2DM. Several randomized controlled trials have shown improved clinical outcomes of SGLT2 inhibitors as monotherapy and as an add-on to oral and insulin therapy [16]. Moreover, the recent clinical trials showed that use of SGLT2i compared with other glucose-lowering drugs was associated with reduced risk for cardiovascular disease morbidity and mortality in adults with T2DM [17]. However, there is a paucity of real-world studies of this novel evaluating similar outcomes especially in Asian patients.

Our findings demonstrated that the effectiveness of SGLT2i in a real-world setting was comparable with results from clinical trials which showed the mean reduction in HbA_1c_ ranged from 0.3 to 1.2% and averaged weight loss 1.5–3.0 kg [18]. Larger reductions in HbA_1c_ were generally seen with higher doses of SGLT2i and among patients with higher baseline HbA_1c_ values. Our results confirmed long-term durability of glycemic control with SGLT2i in not only monotherapy, but also add-on studies with other oral anti-diabetic drugs and/or insulin treatment. However, weight loss became evident early after 6 weeks then reached slightly rebounds after 24 weeks until the end of follow-up at median time of 16 months. Interestingly, our results showed that the individual weight reduction response to SGLT2i differed considerably from one patient to another. Theoretically, SGLT2i causes urinary glucose loss at approximately 80–100 g of glucose per day which translates into a loss of of approximately 320–400 kcal per day [19]. However, the observed weight loss from actual use of SGLT2i was consistently much lower (only 2–4 kg) than that predicted by calorie loss through renal glucosuria. An adaptive increase in food intake had been suggested to explain this difference in weight reduction [20]. In a meta-analysis of randomized control trials for 1–2 years duration of SGLT2i usage revealed that weight reduction were slightly more pronounced at 2 years (− 3.6 to − 2.3 kg) compared with at 1 year (− 2.6 to − 2.4) at 1 year and these differences were even greater when compared with sulfonylureas or thaizolidinediones (weighted mean difference − 5.1 kg at 2 years) [18]. High rate uses of thaizolidinediones and insulin in our long-standing diabetic patients might also affect the less predicted weight reduction from SGLT2i. Weak correlation between HbA_1c_ reduction and weight loss was observed in our observational study same as the recent pool studies study of ipraglifozin in Japanese patients [21]. Therefore, comparison of the long-term effect in weight reduction from SGLT2i in Asian patients and Caucasian patients should be further explored in the future studies.

Since the results of the randomized control trials and an observational study showed that the SGLT2i reduced the incidence of cardiovascular events in patients with type 2 diabetes and established cardiovascular disease, there has been great interest in increasing use of this promising medication in high risk patients with diabetes. Reduced cardiovascular risk appeared to be largely independent glucose-lowering effects and attention is drawn to proposed mechanism of SGLT2i as a metabolic modulator promoting improved cardiac substrate use and overall energetic in cardiac myocytes [17]. However, our real-world setting showed that only 10% of prescribed patients had a history of cardiovascular disease which was consistent with the recent real-world study from United Kingdom [22], and use of SGLT2i had the neutral effects in major adverse cardiovascular events at the median time of follow-up 16 months. Whether this novel class of anti-diabetic agent will lower mortality rates in patients without a history of cardiovascular disease remained to be seen and ongoing clinical trials are eagerly awaited before expanding uses of SGLT2i as a preventive treatment strategy [23].

Of particular interest in our observational study was the combination of SGLT2i plus dipeptidyl peptidase-4 inhibitors (DPP-4i) had a synergistic effect and lead to better glycemic control when compared with added SGLT2i in patients without DPP-4i. Because the pathogenesis of T2DM is complex and involves multiple organs, the use of combination anti-diabetic drugs with different mechanisms of action has the advantage of preventing compensatory mechanisms. While the glucosuric effect of SGLT2i is accompanied by an increased rate of endogenous glucose and glucagon production [24], DPP-4i inhibits glucagon secretion, thus reducing endogenous glucagon production [25]. This additive effect of HbA_1c_ reduction by the addition of SGLT2i to DPP-4i had been demonstrated in clinical trials and likely to be increasingly incorporated into clinical practice [26, 27]. Our study suggested the synergistic effect of this combination drugs. However, selection bias when prescribing diabetic medications from the non-randomized nature of retrospective observational study could also limit this observation. Long-term benefits on beta-cells function, durability, and reduction of complications of this combination would be required in order to provide better assessment of their cost-effectiveness.

Sodium-glucose co-transporter 2 inhibitors pharmacology and reported adverse events should be understood in details before prescribing SGLT2i. It should also be accompanied by appropriate medication counseling especially in elderly patients who are prone to side effects from this novel medication [28]. In this study, evidence suggestive of genital mycotic infections and urinary tract infection was reported in less than 5% of patients and rarely led to discontinuation of medication. These outcomes suggested that SGLT2i is safe for patients in hot climates including Thailand. Even though the recent reported from post hoc analysis of canagliflozin showed higher rate of urinary tract infection in hot climate countries when compared with other areas (7.1% vs. 3.3%), most infections were generally well tolerated and responded to the standard treatments [29]. More common adverse events but neglected concerns would be events linked to volume depletion and hemoconcentration (reflected by increased hemoglobin and hematocrit) observed during SGLT2i treatment. Even though our study showed both efficacy and safety of SGLT2i in elderly patients were consistent with what was observed in the younger patients. Clinicians must take great care when prescribing this medication to frail elderly patients who are vulnerable to dehydration, postural hypotension, and dizziness. Also, special caution should be observed in patients on diuretics. Another serious well-known adverse event from SGLT2i is diabetic ketoacidosis (DKA) which repeatedly reported from post-marketing phase worldwide [30]. The US Food and Drug Administration (FDA) was the first organization to issue this warning in May 2015 [31], shortly followed by a report from the European Medicines Agency (EMA) in June 2015 [32]. Most patients with DKA have a precipitating cause, which includes infections, surgery, dehydration, or insulin dose reduction. Some patients have levels of glu-cose that are lower than what is typically seen in DKA (euglycemic DKA), treating clinicians should keep a high index of suspicion for DKA in patients treated with SGLT2i. Low-carbohydrate intake also had been recognized recently to precipitate DKA from increasing in ketone body formation when SGLT2i was used [33, 34]. In Thailand, the average of carbohydrate intake from daily diet was 210 g/day (55.6% of total energy) [35]. Therefore, it would be emphasized that patients who are taking SGLT2i should not follow a strict low-carbohydrate diet (carbohydrate less than 130 g/day) for weight reduction. As data and clinical experience accrue regarding adverse events from SGLT2i, long-term post-marketing surveillance studies to weigh the risks and benefits are essential.

To the best of our knowledge, this is the first long-term observational study of SGLT2i in Thai patients which reflect actual clinical practice by diabetologists. We demonstrated the durability of glycemic control from SGTL2i up to 18 months but less effectiveness in the observed weight loss from this medication. This highlights the advantage of adding SGLT2i in mainly long-standing duration of diabetes to improve glycemic control as reported from clinical trials and showed the synergistic effect in patients who were already on DPP4i. This real-world study plays an important role in the evaluation of treatment patterns and health outcomes and could supplement knowledge gained from randomized control trials. Because diabetes treatment guidelines have changed over time from initial lifestyle modification to emphasizing early drug treatments, the promising SGLT2i has a potential to incorporate into early stage of diabetes management if ongoing trials also demonstrate class effect from SGLT2i in reduction of cardiovascular events in general diabetic patients [36]. The findings of this study should be also considered in light of some limitations. First, it is a retrospective cohort observational study and as such is prone to the limitations inherent to this study design. ‘Real-world’ patients may differ in several ways that may affect the ability to achieve glycemic control and responded to weight reduction. Second, medication adherence could not be assessed because database only measured filled prescriptions. Third, at the time of study period, more than 90% of patients had been prescribed dapaglifozin as it was the first SGLT2i available in Thailand. The different SGLT2i had slightly different pharmacokinetic effects as assessed by different rates of urinary glucose excretion. However, previous clinical trials demonstrated comparable efficacy between each type of SGLT2i [37].

In conclusion, this real-world study demonstrated the diversity of recent SGLT2i usages with mainly prescribed in long-standing diabetes as a ‘last resort’ therapy before insulin treatment. The durability of glycemic control was consistent with reported from clinical trials but less observed weight loss which tended to rebounded after the first 6 months. Due to the side effects of genital and urinary tract infections, volume depletion and hemoconcentration, careful patients selection accompanied by appropriate realistic treatment goal counseling are warranted. Further studies should be done towards a better understanding of the benefit to risk ratio of treatment with SGLT2 inhibitors, a unique and anticipated anti-diabetic agent.

## Abbreviations

SGLT2i: sodium-glucose co-transporter 2 inhibitors
T2DM: type 2 diabetes mellitus
DPP4i: dipeptidyl peptidase-4 inhibitors
UTI: urinary tract infection
DKA: diabetic ketoacidosis
